# Preliminary Screening for Selected Viral Pathogens in Red Fox Carcasses from Pisa Province, Italy

**DOI:** 10.3390/pathogens15080873

**Published:** 2026-08-20

**Authors:** Mario Forzan, Gemma Banducci, Maurizio Mazzei, Joel Soares Filipe, Ranieri Verin, Matteo Senese, Dania Cingottini, Micaela Sgorbini, Francesca Parisi

**Affiliations:** 1 Department of Veterinary Sciences, University of Pisa, 56124 Pisa, Italy; mario.forzan@unipi.it (M.F.); g.banducci1@studenti.unipi.it (G.B.); maurizio.mazzei@unipi.it (M.M.); joel.soares@unimi.it (J.S.F.); ranieri.verin@unipi.it (R.V.); micaela.sgorbini@unipi.it (M.S.); francesca.parisi@unipi.it (F.P.); 2 Istituto Zooprofilattico Sperimentale del Lazio e della Toscana “M. Aleandri”, 56124 Pisa, Italy; matteo.senese@izslt.it

**Keywords:** red fox, *Vulpes vulpes*, wildlife diseases, viral pathogens, wildlife surveillance, One Health

## Abstract

Wildlife surveillance contributes to the detection of pathogens of veterinary and zoonotic importance. The red fox (*Vulpes vulpes*), which is well adapted to human-modified environments, is frequently included in wildlife pathogen surveillance. This preliminary study screened 20 opportunistically collected red fox carcasses from three neighboring areas of Pisa Province, Italy, for selected viral pathogens, including hepatitis E virus (HEV), canine adenovirus (CAdV), canine circovirus (CanineCV), canine parvovirus (CPV), canine coronavirus (CCoV), and canine bocavirus (CBoV), using an integrated serological, molecular, histopathological, and immunohistochemical approach. Five of 20 serum samples showed ELISA reactivity above the applied positive threshold, while two yielded equivocal results. One fecal sample produced an amplification product of the expected size in the HEV nested RT-PCR assay, but Sanger sequencing did not confirm HEV identity. Sequence-confirmed molecular analysis identified CAdV-1 in the liver and CCoV in the feces of the same fox, whereas CPV, CanineCV, and CBoV were not detected. Histopathological findings were nonspecific, and HEV and CAdV immunohistochemistry yielded negative results. These findings represent limited local observations from an opportunistically collected sample and may help inform the design of larger, systematically sampled investigations in red foxes.

## 1. Introduction

Wildlife plays an increasingly important role in the epidemiology of infectious diseases affecting both animals and humans. More than half of emerging infectious diseases are zoonotic, and many originate from wildlife reservoirs or involve wildlife hosts during their transmission cycles. Consequently, wildlife health surveillance has become a fundamental component of the One Health approach, which recognizes the close interconnection between human, animal, and environmental health and promotes integrated surveillance strategies for infectious diseases [1,2,3,4].

Among European wild carnivores, the red fox (*Vulpes vulpes*) is considered a particularly valuable species for pathogen surveillance because of its wide geographical distribution, remarkable ecological adaptability, and increasing presence in peri-urban and urban environments [5,6,7]. These ecological characteristics may increase opportunities for contact with domestic animals and humans and support the inclusion of red foxes in wildlife pathogen surveillance. Previous studies have demonstrated that red foxes can harbour a variety of viral pathogens of veterinary and zoonotic interest, including canine adenovirus (CAdV), canine parvovirus (CPV), canine coronavirus (CCoV), canine circovirus (CanineCV), canine bocavirus (CBoV), and hepatitis E virus (HEV) [8,9,10,11,12,13,14]. However, the epidemiological significance of these findings often remains difficult to interpret because most investigations rely on opportunistically collected animals and employ different combinations of serological, molecular, or pathological techniques. Interpretation of pathogen detection in wildlife is further complicated by several methodological challenges. Opportunistic sampling frequently results in small and potentially biased study populations, while post-mortem changes, variable carcass preservation, and differences in sample quality may influence both molecular and pathological investigations. For these reasons, combining complementary diagnostic approaches, including serology, molecular assays, histopathology, and immunohistochemistry, may provide complementary information, although the contribution of each method may differ depending on the pathogen and sample quality.

The viral panel investigated in this study was selected on the basis of three main criteria: previous detection or investigation in red foxes and other European wild carnivores, veterinary or zoonotic relevance, and the availability of published diagnostic assays applicable to the biological matrices collected. HEV was included because of its zoonotic relevance and previous reports of HEV or HEV-like viruses in red foxes, whereas CAdV, CPV, CCoV, CanineCV, and CBoV were selected as canine-associated viruses previously reported in wild carnivores and potentially relevant at the wildlife–domestic animal interface [8,9,10,11,12,13,14]. The selected viruses therefore represent a targeted, non-exhaustive panel consistent with the descriptive and exploratory scope of the study. Within this context, the present study aimed to perform a preliminary targeted screening of opportunistically collected red fox carcasses from three neighboring areas of Pisa Province using serological, molecular, histopathological, and immunohistochemical approaches. Rather than estimating pathogen prevalence or inferring transmission dynamics, the study was designed to provide a descriptive assessment of the examined animals and to evaluate the information contributed by the different diagnostic approaches.

## 2. Materials and Method

### 2.1. Study Area and Sample Collection

A total of 20 red foxes were included in this study. Sixteen carcasses were obtained during the 2023–2024 hunting season within wildlife management programs authorized by the Tuscany Region (Regional Decree No. 815/2022). Four additional animals consisted of free-ranging red foxes admitted to the Veterinary Teaching Hospital of the University of Pisa following road traffic accidents; these animals either died spontaneously or were humanely euthanized. The carcasses originated from three neighboring areas within the province of Pisa (Central Italy): Navacchio (*n* = 8), San Miniato–Collebrunacchi (*n* = 8), and Latignano (*n* = 4). These locations represent the geographical origin of the animals, irrespective of whether carcasses originated from wildlife management activities or road traffic accidents. Because these sites are geographically close and belong to a relatively homogeneous local context, spatial comparisons were considered descriptive only and were not interpreted as evidence of distinct epidemiological patterns. Site-specific data on habitat composition, hunting pressure, and other ecological variables were not systematically collected; therefore, no ecological comparisons among the sampling locations were performed. After collection, all carcasses were stored at −20 °C until necropsy. For each animal, sex, estimated age (based on dental wear and cranial suture closure), body weight, and geographical origin were recorded. The study was designed as a preliminary descriptive investigation based on opportunistically collected carcasses. Data were summarized using absolute counts, descriptive percentages, and observed ranges. Exact two-sided 95% confidence intervals for the principal proportions were calculated using the Clopper–Pearson method in Python version 3.13.5 with SciPy version 1.17.0. No hypothesis testing, group comparisons, multivariable analyses, or other inferential procedures were performed because of the limited sample size and opportunistic sampling design. Given the non-probability sampling strategy, the confidence intervals were not interpreted as population-level prevalence estimates.

### 2.2. Necropsy and Sample Processing

Necropsies were performed between May and July 2024 at the Department of Veterinary Sciences, University of Pisa. A complete post-mortem examination was conducted, including external inspection and evaluation of thoracic and abdominal organs. The following samples were collected: mandibular lymph nodes, liver, and feces (rectal ampulla). Blood samples were collected via intracardiac puncture in all animals, and, when feasible, via cavernous sinus puncture to obtain serum of improved quality.

Serum samples were separated and stored at −20 °C until serological analysis. Each tissue sample was divided for molecular, histopathological, and immunohistochemical analyses. Samples intended for molecular investigations were stored at −20 °C, whereas parallel tissue sections were fixed in 10% neutral buffered formalin (pH 7.4).

### 2.3. Serological Analysis

Serological testing for antibodies against hepatitis E virus (HEV) was performed using a commercial double-antigen ELISA kit (HEV Ab Version ULTRA, DIA.PRO, Milan, Italy) designed for the detection of total anti-HEV antibodies, including IgA, IgM, and IgG. The assay uses HEV-specific synthetic antigens as both capture and peroxidase-labelled detection reagents and therefore does not rely on a species-specific anti-immunoglobulin conjugate. According to the manufacturer, this configuration permits its application to serum or plasma from non-human hosts in zoonotic studies. Manufacturer-provided positive and negative controls were included in the analytical run and evaluated according to the assay validity criteria. Absorbance was measured at 450 nm, and results were expressed as the ratio between sample optical density and the calculated cut-off (S/CO). In the absence of a red fox-specific reference panel, the manufacturer’s S/CO thresholds were used for exploratory classification of the samples. However, the diagnostic performance data reported by the manufacturer were obtained from human samples, and formal validation in red foxes has not been reported. No red fox- or canid-derived reference sera of known HEV status or independent cross-reactivity panel were available. Therefore, the species-specific sensitivity, specificity, and optimal cut-off of the assay could not be determined. The resulting classifications were consequently interpreted as exploratory ELISA reactivity rather than as definitive evidence of HEV infection or previous exposure.

### 2.4. Nucleic Acid Extraction

DNA was extracted from liver and fecal samples using the Monarch Genomic DNA Purification Kit (New England Biolabs, Ipswich, MA, USA), following the manufacturer’s instructions. RNA extraction from liver and fecal samples was performed using the QIAamp Viral RNA Mini Kit (QIAGEN, Hilden, Germany). Extracted DNA and RNA were aliquoted and stored at −20 °C and −80 °C, respectively, until molecular analysis.

### 2.5. Molecular Detection of Viral Pathogens

Molecular detection of viral pathogens was performed using polymerase chain reaction (PCR)-based assays targeting selected DNA and RNA viruses. Conventional PCR assays were used for the detection of canine adenovirus (CAdV), canine parvovirus (CPV), canine bocavirus (CBoV), and canine circovirus (CanineCV), whereas nested RT-PCR assays were employed for hepatitis E virus (HEV) and canine coronavirus (CCoV). The CAdV assay was performed according to Hu et al. (2001) [15], allowing differentiation between CAdV-1 and CAdV-2 based on the size of the amplification products. Broad-range primers targeting conserved genomic regions were employed where appropriate to maximize the detection of genetically diverse viral strains. Primer sequences are reported in Table 1, whereas assay-specific technical characteristics are summarized in Supplementary Table S1. All PCR and nested RT-PCR assays were performed using a common thermal profile consisting of initial denaturation at 95 °C for 5 min, followed by 35 amplification cycles at 95 °C for 30 s, assay-specific annealing for 30 s, and extension at 72 °C for 45 s, with a final extension at 72 °C for 7 min. Annealing temperatures were 50 °C for HEV, 58 °C for CAdV, 55 °C for CPV, 60 °C for CBoV and CanineCV, 48 °C for CCoV, and 60 °C for the red fox 18S rRNA gene. The expected amplification products were 325 bp for HEV, 508 bp for CAdV-1 and 1030 bp for CAdV-2, 98 bp for CPV, 290 bp for CBoV, 129 bp for CanineCV, 602 bp for CCoV, and 228 bp for the 18S rRNA gene. Template volumes were 3 µL for RNA assays and 2.5 µL for DNA assays; for nested RT-PCR assays, 1.5 µL of the first-round amplification product was used in the second round. Each amplification run included a no-template negative control consisting of molecular-grade water. Selection of biological matrices was based on the known tissue tropism and principal sites of viral replication for each pathogen. Liver samples were analyzed for CAdV because canine adenovirus type 1 primarily targets hepatocytes during systemic infection, and for HEV because the liver represents the principal site of viral replication. Fecal samples were selected for CPV, CanineCV, CBoV, CCoV, and HEV because these viruses are commonly shed through the gastrointestinal tract and feces represent the biological matrix most frequently used for molecular detection in wildlife surveillance. Prior to viral DNA screening, the quality and amplifiability of extracted DNA were assessed by amplification of the red fox 18S rRNA gene using the primers described by Rolland-Turner et al. (2006) [16]. RNA extracts were evaluated spectrophotometrically using a NanoDrop instrument to assess nucleic acid concentration and purity prior to downstream analyses. However, no endogenous RNA reference target or external RNA extraction/process control was included in the molecular workflow. Therefore, while basic RNA quality parameters were assessed, RNA integrity and extraction efficiency were not independently verified.

Samples yielding the expected amplification product were considered suitable for downstream DNA-based molecular analyses. PCR products showing a single amplicon of the expected size after agarose gel electrophoresis were purified and subjected to bidirectional Sanger sequencing (BMR Genomics, Padua, Italy). Sequence chromatograms were visually inspected, and low-quality bases at both sequence ends were trimmed before generating consensus sequences from forward and reverse reads. Consequently, the length of the analyzed consensus sequences may be shorter than the expected PCR amplicon size. Viral identity was confirmed by comparison with reference sequences available in the GenBank database using the NCBI Web BLAST (blastn) algorithm. For the HEV nested RT-PCR, amplification products of the expected size were not considered evidence of HEV detection unless viral identity was confirmed by sequencing. When an HEV-compatible amplification product was obtained, the assay was repeated using the same RNA extract and the product was subjected to bidirectional Sanger sequencing. Products for which an interpretable HEV-derived sequence could not be obtained were classified as unconfirmed amplifications. To minimize the risk of carry-over contamination, reagent preparation, nucleic acid extraction, amplification, and post-PCR procedures were performed in physically separated laboratory areas (no-copy, low-copy, and high-copy) using dedicated equipment, dedicated pipettes, and aerosol-resistant filter tips. Primers used for molecular detection of viral pathogens and amplification of the red fox housekeeping gene are listed in Table 1.

### 2.6. Histopathological and Immunohistochemical Analysis

FFPE tissue sections (4 μm) were stained with hematoxylin and eosin (H&E) for routine histopathological evaluation. Lesions including inflammatory infiltrates, edema, congestion, and parasitic changes were systematically recorded. Histopathology and immunohistochemistry were performed to assess whether serological or molecular findings were associated with detectable tissue lesions or viral antigen and to identify concurrent pathological conditions that could influence interpretation of the virological results. Additional sections prepared for immunohistochemistry (IHC) were mounted on positively charged slides (Menzel-Gläser Superfrost Plus; Thermo Fisher Scientific, Waltham, MA, USA) and dried at 37 °C for 24 h. Immunohistochemistry was performed using a labeled streptavidin-biotin (LSAB) method. After heat-induced antigen retrieval in sodium citrate buffer (pH 6.0), endogenous peroxidase activity and non-specific binding were blocked using BLOXALL Blocking Solution SP-6000 (Vector Laboratories, Newark, CA, USA) and UltraVision Protein Block (Thermo Fisher Scientific, USA), respectively. Sections were incubated with primary antibodies overnight at room temperature. To evaluate tissue antigenicity, lymph node sections were incubated with a polyclonal rabbit anti-CD3 antibody (Dako, Glostrup, Denmark; dilution 1:200) and a polyclonal rabbit anti-CD20 antibody (dilution 1:100) (Thermo Fisher Scientific, Waltham, MA, USA). Liver sections were incubated with a monoclonal mouse anti-HEV antibody (clone 4B2, dilution 1:100) (Chemicon International, Temecula, CA, USA) and a polyclonal goat antibody showing cross-reactivity against several adenoviruses (dilution 1:1600) (AbD Serotec, Kidlington, Oxford, UK). Sections were subsequently incubated with a biotinylated secondary antibody and a streptavidin-peroxidase complex (Vector Laboratories, USA). Immunoreactivity was visualized using 3,3′-diaminobenzidine (DAB), and slides were counterstained with hematoxylin. Canine lymph node sections were used as positive controls for CD3 and CD20 staining. Liver sections from a PCR-confirmed HEV-positive rabbit and a PCR-confirmed cAdV-positive dog were used as positive controls for the respective markers. Negative controls were obtained by replacing the primary antibodies with irrelevant isotype-matched antibodies. Slides were then examined under light microscopy.

### 2.7. Ethical Considerations

No animals were killed specifically for this study. Samples were obtained either from red foxes legally culled within wildlife management programs authorized by the Tuscany Region (Regional Decree No. 815/2022) or from free-ranging injured foxes admitted to the Veterinary Teaching Hospital of the University of Pisa following road traffic accidents that subsequently died spontaneously or were humanely euthanized for welfare reasons.

## 3. Results

### 3.1. Sample Characteristics

The study population comprised 20 foxes, including 9 males and 11 females. Most animals (17/20) were classified as young adults (1–2 years), whereas three were estimated to be 2–3 years old. Body weight ranged from 3.6 to 8.8 kg, and no evident signs of poor general condition were observed during post-mortem examination. Individual animal-level demographic, sampling, serological, molecular, sequencing, immunohistochemical, and pathological data are provided in Supplementary Table S2. An integrated summary of the principal findings for each investigated pathogen is provided in Table 2.

### 3.2. Serological Findings

According to the manufacturer’s S/CO thresholds, which were used for exploratory classification, 5 of 20 foxes showed ELISA reactivity above the applied positive threshold (25.0%; 95% CI: 8.7–49.1%), while two additional animals yielded equivocal results (10.0%; 95% CI: 1.2–31.7%). Because the assay has not been formally validated in red foxes and no species-specific reference panel was available, these classifications were interpreted as exploratory ELISA reactivity rather than as definitive evidence of HEV infection or previous exposure.

### 3.3. Molecular Detection Results

One fecal sample yielded an amplification product compatible with the expected size in the HEV nested RT-PCR assay. The assay was repeated several times using the same RNA extract. However, bidirectional Sanger sequencing did not yield an interpretable target-derived sequence beyond the primer-associated regions, and HEV identity could therefore not be established. The product was consequently classified as an unconfirmed amplification of undetermined identity and was excluded from the positive molecular findings. Sequence-confirmed molecular analysis identified CAdV-1 in the liver and CCoV in the feces of the same fox (1/20; 5.0%; 95% CI: 0.1–24.9%). The expected CAdV-1 PCR amplicon was 508 bp. After trimming low-quality terminal bases from the bidirectional Sanger reads, a 470-bp high-quality consensus sequence was obtained and used for BLAST analysis and GenBank submission. The difference between the expected amplicon size and the consensus length reflects sequence-quality trimming and does not indicate a genomic deletion. The CAdV-1 consensus sequence showed 99.77% nucleotide identity and 99% query coverage with GenBank sequence KT853096, whereas the CCoV sequence showed 99.75% nucleotide identity and 100% query coverage with sequence ON834692. The sequences generated in this study were deposited in GenBank under accession numbers PZ577540 and PZ577541, respectively. Sequencing and BLASTn results are reported in Table 3. No molecular detection of canine parvovirus (CPV), canine circovirus (CanineCV), or canine bocavirus (CBoV) was obtained in the examined fecal samples. An integrated summary of the molecular and other diagnostic findings for all investigated pathogens is provided in Table 2. Sequence identity, query coverage, closest reference sequences, and GenBank accession numbers are reported in Table 3.

### 3.4. Histopathological and Immunohistochemical Findings

Pulmonary edema was a common finding, observed in 9 out of 20 examined samples, and was consistently associated with vascular congestion. Pulmonary lesions were identified in 10 of the 20 examined cases: five animals showed mixed pneumonia, whereas five cases were characterized by parasitic pneumonia associated with the presence of pulmonary nematodes. In one additional case, inflammatory changes were observed in the liver. Follicular hyperplasia of the lymph nodes, indicative of reactive lymphoid tissue, was detected in 6 out of 20 animals. Inflammatory infiltrates were identified in 6 of the 20 examined cases: in five animals the lesions were localized in the lungs and associated with the presence of pulmonary nematodes, whereas in one case inflammatory changes were observed in the liver. Immunohistochemical analysis of lymph node sections revealed membranous immunoreactivity for anti-CD3 antibody within the paracortical areas and for anti-CD20 antibody within cortical follicular structures. Positive staining was observed in all examined samples. Immunohistochemical investigation performed on liver sections using anti-HEV and anti-cAdV antibodies yielded negative results in all samples. Overall, the pathological findings were nonspecific or attributable to concurrent conditions and did not provide pathogen-specific confirmation of the serological or molecular results.

## 4. Discussion

The present study provides a limited descriptive assessment of selected viral pathogens in opportunistically collected red fox carcasses originating from three neighboring areas of Pisa Province. Given the small sample size and sampling strategy, the findings are restricted to the examined animals and were not intended to support population-level or broader epidemiological conclusions. The results indicate serological reactivity to HEV or HEV-related antigens in a subset of the examined foxes, whereas no sequence-confirmed molecular evidence of HEV infection was obtained. Previous European studies have produced markedly different serological findings in red foxes. In Brandenburg, Germany, Eiden et al. examined 880 fox cavity transudates collected between 1993 and 2012 and reported high HEV-related antibody reactivity, whereas only one sample yielded a sequence assigned to *Orthohepevirus C* [14]. In contrast, Prpić et al. detected neither anti-HEV antibodies nor HEV RNA in Croatian red foxes; serological testing in that study was performed on muscle extracts using an indirect multispecies ELISA [14,22,23]. The proportion of ELISA-reactive serum samples observed in the present study (5/20; 25.0%, 95% CI: 8.7–49.1%) cannot be directly compared with these findings. The studies differed substantially in biological matrix, assay configuration and antigens, cut-off determination, confirmatory procedures, sample size, geographical coverage, and sampling period. In particular, the German study included additional strip immunoassay and Western blot evaluation of selected reactive samples, whereas the assay used in the present study has not been formally validated in red foxes. Therefore, the differences among studies cannot be attributed to geographical variation alone and may partly reflect methodological and sampling heterogeneity.

The discordance between serological reactivity and the absence of sequence-confirmed HEV RNA may have several, non-mutually exclusive explanations. Animals showing ELISA reactivity may have experienced previous exposure followed by viral clearance, while transient, intermittent, or low-level shedding may have reduced the likelihood of molecular detection at the time of sampling. The double-antigen configuration of the HEV-Ab ULTRA assay does not require a species-specific anti-immunoglobulin conjugate and supports its exploratory application to samples from non-human hosts. Nevertheless, the diagnostic performance reported by the manufacturer was established using human samples and cannot be directly transferred to red foxes. In addition, no red fox- or canid-derived reference sera of known HEV status and no independent cross-reactivity panel were available. Consequently, the sensitivity, specificity, and optimal cut-off of the assay in red foxes remain unknown. Although interference from antibodies directed against unrelated canine viruses is considered less likely because the assay requires binding to HEV-specific synthetic antigens, nonspecific matrix effects or cross-reactivity with antigenically related hepeviruses cannot be excluded. The reproducibility of the HEV-compatible amplification in repeated assays using the same RNA extract indicates that amplification occurred consistently from that extract, but does not establish target specificity. Because sequencing did not yield an interpretable target-derived sequence, nonspecific amplification or amplification of an unintended template cannot be excluded. In addition, carcass preservation and the absence of an endogenous RNA control or external process control may have reduced the sensitivity of the RT-PCR assay. Because these possibilities cannot be distinguished with the available data, the ELISA results should be interpreted as antibody reactivity to HEV or HEV-related antigens, whereas the lack of sequence-confirmed RNA does not establish active infection, exclude previous exposure, or support conclusions regarding HEV circulation in the examined foxes. Sequence-confirmed molecular detection identified CAdV-1 in the liver and CCoV in feces of a single fox. These findings are consistent with previous detections of CAdV-1 in free-ranging red foxes and other wild carnivores in Italy and elsewhere in Europe [8,9,12,24]. However, caution must be exercised in interpreting these data; the detection of CAdV-1 and CCoV in a single individual does not allow conclusions regarding pathogen maintenance, transmission dynamics, or reservoir competence. Within the limitations of the present study, these findings should be interpreted as occasional sequence-confirmed detection of canine-associated viral nucleic acids in a local red fox population. Although CAdV-1 and CCoV were sequence-confirmed in the same fox, this finding should be interpreted as concurrent molecular detection rather than as evidence of a clinically relevant co-infection. No ante-mortem clinical history was available, and post-mortem examination did not identify a specific lesion pattern attributable to either virus. CAdV-1 was detected in the liver, but CAdV immunohistochemistry was negative. CCoV was detected in feces, whereas no intestinal tissue or CCoV-specific immunohistochemical assay was available for direct pathological correlation. Consequently, the contribution of either virus to disease in this animal, as well as any interaction capable of increasing pathogenicity, could not be assessed. The clinical and ecological significance of the concurrent detection therefore remains undetermined. No molecular evidence of CPV, CanineCV, or CBoV was detected in the examined samples. This result should not be interpreted as evidence of absence of these viruses in the local fox population, given the limited sample size, opportunistic sampling strategy, and the use of selected biological matrices. Temporal variation in viral shedding, low viral loads, and sample preservation may all have influenced molecular detection. Histopathology and immunohistochemistry did not provide pathogen-specific confirmation of the serological or molecular findings. This lack of concordance may partly reflect the fact that the different methods assessed distinct biological targets. Serology measured antibody reactivity, molecular assays detected viral nucleic acids in selected biological matrices, histopathology evaluated tissue lesions, and immunohistochemistry required detectable viral antigen within the examined tissue sections. Therefore, agreement among these approaches was not necessarily expected, particularly in the presence of previous exposure, low viral burden, transient infection, focal antigen distribution, or limited antigen expression.

The most frequent lesions, including pulmonary edema, vascular congestion, pulmonary lesions, some associated with pulmonary nematodes, and reactive lymphoid hyperplasia, were nonspecific or attributable to concurrent conditions. For HEV, the absence of immunoreactivity was consistent with the lack of sequence-confirmed molecular detection. For CAdV-1, the negative immunostaining in the PCR-positive liver may have resulted from low viral burden, focal or uneven antigen distribution, post-mortem or freeze–thaw-related antigen degradation, or the lower analytical sensitivity of immunohistochemistry compared with PCR. In addition, CCoV was detected in feces, whereas no intestinal tissue or CCoV-specific immunohistochemical assay was available for direct pathological correlation. Histopathology and immunohistochemistry should therefore be interpreted as complementary assessments of tissue condition and concurrent disease rather than as confirmatory evidence of infection.

Several limitations should be considered when interpreting the results. First, the study included only 20 foxes collected opportunistically from geographically close areas; therefore, the data cannot be used to estimate pathogen prevalence at the population level or to assess statistical associations between host characteristics and pathogen detection. Second, the study population included foxes obtained through wildlife management activities and animals admitted after road traffic accidents. Although this introduced some heterogeneity in carcass source, all animals originated from the same restricted geographical context within Pisa Province and were collected within the same sampling period. The study was not designed to compare the two groups, and the mixed origin of the carcasses does not substantially alter the local and temporal scope of the findings. The main relevant difference concerned sample preservation, as animals admitted to the Veterinary Teaching Hospital were examined under more controlled conditions and were less exposed to the potential effects of prolonged carcass storage. Conversely, freezing and post-mortem changes in hunted carcasses may have affected tissue morphology, antigen preservation, and RNA integrity. Given the limited number of road-traffic-accident cases, no formal comparison between the two sources was performed. These aspects should be considered when interpreting findings from passive and opportunistic wildlife surveillance [25]. Sample preservation represents an additional limitation. Although storage at −20 °C is not expected to substantially affect antibody detection when repeated freeze–thaw cycles are avoided, tissue-based analyses such as histopathology and immunohistochemistry are more susceptible to autolysis and freezing artifacts. For molecular investigations, amplification of the red fox 18S rRNA gene confirmed the amplifiability of extracted DNA. However, amplification of the red fox 18S rRNA gene assessed only DNA amplifiability and did not evaluate the integrity of the RNA extracts. Because no endogenous RNA target or external process control was included, RNA degradation or extraction inefficiency cannot be excluded and may have reduced the sensitivity of the HEV and CCoV RT-PCR assays. The sequence-confirmed CCoV result indicates that amplifiable viral RNA was present in at least one sample, but it does not validate the quality of all RNA extracts. Consequently, negative RT-PCR findings, particularly for HEV, should be interpreted cautiously and should not be regarded as evidence of viral absence. Future studies should include an endogenous RNA reference gene or an external extraction/process control to verify RNA integrity and assay performance. In the present study, the individual diagnostic approaches did not contribute equally to the final interpretation. Sequence-confirmed molecular detection represented the primary evidence supporting pathogen identification, whereas histopathology and immunohistochemistry did not contribute directly to pathogen identification but were useful for describing tissue alterations and excluding alternative pathological interpretations.

Nevertheless, considering findings from different diagnostic levels together allowed the molecular and serological results to be interpreted in relation to tissue-level observations and the limitations of the available samples, without implying equivalent diagnostic value for each method. Although the study was conceived within a One Health context, the present dataset does not allow conclusions regarding interspecies transmission, pathogen circulation, or the broader role of red foxes at the wildlife–domestic animal–human interface [1,2]. The combined diagnostic workflow nevertheless permitted the findings and their limitations to be documented at serological, molecular, and tissue levels. Its applicability and diagnostic value should be further evaluated in larger studies based on systematic sampling and standardized sample preservation [25].

The principal contribution of the present study is the provision of an animal-level, locally defined dataset in which serological, molecular, sequencing, and pathological findings were evaluated together. This allowed a clear distinction between HEV serological reactivity, an unconfirmed HEV-compatible RT-PCR product, and sequence-confirmed CAdV-1 and CCoV detections, while also documenting the contribution of histopathology and immunohistochemistry to the overall interpretation, although no specific pathological correlation with the virological findings was identified in the examined cases. The findings thus add descriptive information from Pisa Province and highlight methodological aspects relevant to the interpretation of opportunistically collected wildlife samples.

Overall, the present findings provide preliminary descriptive observations from the examined animals. Larger studies based on systematic sampling, broader geographical coverage, standardized sample preservation, RNA-specific quality controls, and validated diagnostic assays are required to assess the occurrence and significance of these viruses in red foxes.

## 5. Conclusions

This study provides descriptive observations on selected viral pathogens in opportunistically collected red fox carcasses from Pisa Province. Serological reactivity to HEV or HEV-related antigens was detected in a subset of animals, whereas no sequence-confirmed molecular evidence of HEV infection was obtained. Sequence-confirmed molecular detection identified CAdV-1 in the liver and CCoV in the feces of a single fox, while CPV, CanineCV, and CBoV were not detected in the examined samples.

Sequence-confirmed molecular analysis provided the strongest pathogen-specific evidence in this study. In contrast, histopathology and immunohistochemistry did not provide confirmatory evidence and contributed mainly descriptive information on tissue condition and concurrent pathological findings. The results should therefore be interpreted in the context of the examined animals and should not be extrapolated to the wider red fox population. Larger studies based on systematic sampling, broader geographical coverage, standardized sample preservation, RNA-specific quality controls, and validated diagnostic assays are required to assess the occurrence and significance of these viruses in red foxes.

## Figures and Tables

**Table 1:** Primer sequences used for molecular detection of the investigated viral pathogens and amplification of the red fox 18S rRNA gene. Forward and reverse primer sequences and the corresponding literature references are reported for each PCR and nested RT-PCR assay. Detailed assay characteristics are provided in Supplementary Table S1.

Virus	Primers	Sequence	Reference
Adenovirus (*CAdV 1,2*)	CAdv_fw	CGCGCTGAACATTACTACCTTGTC	[15]
	CAdv_rev	CCTAGAGCACTTCGTGTCCGCTT	
Parvovirus	Can_parvo_FW	ACAAGATAAAAGACGTGGTGTAACTCAA	[17]
	Can_parvo_Rev	CAACCTCAGCTGGTCTCATAATAGT	
Bocavirus	Boca_FW	GCCAGCACNGGNAARACMAA	[18]
	Boca_rev	CATNAGNCAYTCYTCCCACCA	
Circovirus	Canine CV FW	CTGAAAGATAAAGGCCTCTCGCT	[19]
	Canine CV Rev	AGGGGGGTGAACAGGTAAACG	
Coronavirus	Can_Corona_fw	GGKTGGGAYTAYCCKAARTG	[20]
	Can_Corona_rev	TGYTGTSWRCARAAYTCRTG	
HEV	HEV-cs	TCGCGCATCACMTTYTTCCARAA	[21]
	HEV-cas	GCCATGTTCCAGACDGTRTTCCA	
	HEV-csn	TGTGCTCTGTTTGGCCCNTGGTTYCDG	
	HEV-casn	CCAGGCTCACCRGARTGYTTCTTCCA	
18S rRNA	Fox18S_F Fox18S_R	TTCAAGAACGAAAGTCGGAG TGAGTCAAATTAAGCCGCAG	[16]

**Table 2:** Integrated summary of the principal findings for each investigated pathogen.

Pathogen	Biological Sample	Serology	Molecular Findings	Pathological Findings	Overall Interpretation
HEV	Serum, liver, feces	5 reactive	Not sequence-confirmed	NSL;	Serological reactivity;
		2 equivocal		IHC negative	no sequence-confirmed
CAdV-1	Liver	ND	1 sequence-confirmed	NSL;	Sequence-confirmed
				IHC negative	(CAdV-1)
CCoV	Feces	ND	1 sequence-confirmed	NI	Sequence-confirmed
CPV	Feces	ND	Negative	NI	No molecular detection in the examined samples
CanineCV	Feces	ND	Negative	NI	No molecular detection in the examined samples
CBoV	Feces	ND	Negative	NI	No molecular detection in the examined samples

Note: ND: Not Detected; IHC: immunohistochemistry; NSL: Not Specific Lesions; NI: Not Investigated (indicates that the corresponding analytical approach was not performed for that pathogen). For CCoV, no pathogen-specific histopathological or immunohistochemical assessment was performed, and the fecal molecular finding could not be directly correlated with intestinal tissue lesions.

**Table 3:** Sanger sequencing and BLASTn analysis of sequence-confirmed PCR products. Viral identity was assessed by bidirectional Sanger sequencing followed by comparison with reference sequences deposited in the GenBank database. The table reports the GenBank accession number of each sequence generated in this study, biological matrix, amplified genomic target, high-quality trimmed consensus length, closest reference sequence, nucleotide identity, and query coverage. Expected PCR amplicon sizes are reported in Supplementary Table S1. Differences between expected amplicon size and consensus length reflect trimming of low-quality terminal bases and do not indicate genomic deletions.

GenBank Accession Number	Tissue	PCR Target	Consensus Length	Closest Gene Bank Sequence	Identity (%)	Query Cover (%)
PZ577540	Liver	Adenovirus E3 gene	470 bp	Canine adenovirus 1 KT853096	99.77%	99%
PZ577541	Fecal samples	Coronavirus RNA-dependent RNA polymerase	602 bp	Canine coronavirus ON834692	99.75%	100%

## Data Availability

The data presented in this study are available on request from the corresponding author. The sequences generated in this study were deposited in GenBank under accession numbers PZ577540 and PZ577541, respectively.

## Supplementary Materials

The following supporting information can be downloaded at: https://www.mdpi.com/article/10.3390/pathogens15080873/s1, Table S1: Technical characteristics of the PCR and RT-PCR assays used for molecular detection of viral pathogens and housekeeping gene; Table S2: Individual-level demographic, serological, molecular, sequencing, immunohistochemical, and pathological data for the 20 red foxes.

## Abbreviations

The following abbreviations are used in this manuscript:

CPV	Canine parvovirus
CAdV	Canine adenovirus
HEV	Hepatitis E virus
R	Ratio
OD	Optical Density
CO	Cut-off
PCR	Polymerase chain reaction
CBoV	Canine bocavirus
CanineCV	Canine circovirus
CCoV	Canine coronavirus
H&E	Hematoxylin and eosin
IHC	Immunohistochemistry
LSAB	Labeled streptavidin-biotin
DAB	3,3′-diaminobenzidine
VTH	Veterinary teaching hospital
