# Supplementary material for: Preliminary Screening for Selected Viral Pathogens in Red Fox Carcasses from Pisa Province, Italy
Source: Pathogens. 2026 Aug 20;15(8):873. doi: 10.3390/pathogens15080873 (PMC13516290; doi:10.3390/pathogens15080873)
Supplement: Supplementary file 1 [file pathogens-15-00873-s001.zip › pathogens-4478607-supplementary Table S1.pdf]

Supplementary Table S1

Supplementary Table S1. Technical characteristics of the PCR and RT-PCR assays used for molecular detection of viral pathogens and housekeeping gene.

Virus	Biological matrix	Target gene	Assay	Expected amplicon (bp)	Annealing temperature (°C)	Reference
HEV	Liver<br>Feces	ORF1	Nested RT-PCR	325 bp	50°C	Johne et al. (2010) [21]
CAdV	Liver	E3 gene	PCR	508 bp (CAdV-1)<br>1030 bp (CAdV-2)	58°C	Hu et al. (2001) [15]
CPV	Feces	VP2 capsid protein	PCR	98 bp	55°C	Schatzberg et al. (2003) [17]
CBoV	Feces	NS1 gene	PCR	290 bp	60°C	Lau et al. (2012) [18]
CanineCV	Feces	replicase	PCR	129 bp	60°C	Balboni et al. (2021) [19]
CCoV	Feces	RdRp (ORF1b)	Nested RT-PCR	602 bp	48°C	Chu et al. (2011) [20]
18S rRNA	DNA	18S	PCR	228 bp	60°C	Rolland-Turner et al. (2006) [16]

Note. All PCR and nested RT-PCR assays were performed using a common thermal profile consisting of an initial denaturation step, followed by 35 amplification cycles (95°C for 30 s, assay-specific annealing temperature for 30 s, and 72°C for 45 s), and a final extension at 72°C for 7 min. The assay-specific annealing temperatures are reported in the table. Template volume was 3 µL for RNA assays and 2.5 µL for DNA assays. For nested PCR assays, 1.5 µL of the first-round amplification product was used as template. Each PCR and nested RT-PCR run included a no-template negative control (molecular-grade water). Positive amplicons were confirmed by bidirectional Sanger sequencing followed by BLAST analysis against GenBank reference sequences, except for the HEV-compatible amplicon, which failed sequence confirmation and was interpreted as an unconfirmed amplification. PCR setup, nucleic acid extraction, amplification, and post-PCR procedures were performed in physically separated no-copy, low-copy, and high-copy laboratory areas using dedicated equipment, dedicated pipettes, and aerosol-resistant filter tips.
